# Analysis of depression status and influencing factors in middle-aged and elderly patients with chronic diseases

**DOI:** 10.3389/fpsyg.2024.1308397

**Published:** 2024-02-16

**Authors:** Wenjie Lin, Danling Zhang, YiMin Wang, Li Zhang, Jianchuan Yang

**Affiliations:** ^1^Department of Physician, Community Health Service Center of Shangdu Street, Fuzhou, Fujian, China; ^2^Department of Ultrasonography, Fujian Medical University Union Hospital, Fuzhou, China; ^3^Department of Basic Surgery, Fujian Provincial Hospital, Shengli Clinical Medical College of Fujian Medical University, Fuzhou, Fujian, China; ^4^Department of Nephrology, Fujian Provincial Hospital, Shengli Clinical Medical College of Fujian Medical University, Fuzhou, Fujian, China; ^5^Department of Ultrasonography, Fujian Provincial Hospital, Shengli Clinical Medical College of Fujian Medical University, Fuzhou, Fujian, China

**Keywords:** depression, middle and old age, chronic diseases, influencing factors, LASSO logistic model

## Abstract

**Objectives:**

To explore prevalence of depression and its influencing factors in middle-aged and elderly patients with chronic diseases.

**Method:**

Data were extracted from the 2018 China Health and Retirement Tracking Survey (CHARLS) for 6,704 middle-aged and elderly patients ≥45 years with chronic diseases. The influencing variables were selected based on LASSO-logistic regression model, and a nomogram was further drawn to visualize regression results.

**Results:**

Comorbidity between chronic diseases and depression symptoms were detected in 3058 individuals (45.6%). Female, rural, lower education, poor, insomnia, multiple chronic disease, and functional impairment were associated with a higher proportion of depression. Meanwhile, family interaction, intergenerational financial support, social activity intensity, and satisfaction with life can protect against depression.

**Conclusion:**

Depressive symptoms are common in Chinese older adults with chronic diseases. They need regular assessment and intervention, especially those with multiple diseases, female, rural, alone, impaired, poor sleep, or poor economy. These high-risk elders also need family, medical, and social support and care.

## 1 Introduction

Aging is characterized by a decline in the individual’s physical health and a gradual increase in the incidence of chronic diseases, which seriously affects the mental health. Depression is one of the most common but poorly recognized and understood mental illnesses among middle-aged and elderly people (Herrman et al., 2022), may instigate self-harm and suicidal tendency (Li and Ma, 2017). In patient with chronic diseases, the prevalence of major depressive disorder is approximately two-to three-fold higher than in the general population (Almeida et al., 2020).

The association between depression and chronic disease is definite. Physical symptoms such as stroke, blindness, deafness, arthritis, heart disease and chronic lung disease can cause or increased depression symptoms (Moussavi et al., 2007; Clarke and Currie, 2009; Huang et al., 2010; Compare et al., 2013). Recent evidence from the China Health and Retirement Longitudinal Study (CHARLS) also confirmed that, older adults with chronic disease had a high risk of depression (Liu H. et al., 2023). Thus, the comorbidity of chronic diseases with depression calls for more attention, especially in China, with an older population exceeding 200 million.

The comorbidity of depression with chromic medical diseases is associated with deterioration of quality of life, out of pocket expenses, and considerable functional impairment. Healthcare expenditures for chronic diseases can worsen financial condition (Hajat and Stein, 2018), resulting in more likely to suffer depression (Katon et al., 2003; Lue et al., 2010; Conde-Sala et al., 2019). There is strong evidence that cognitive impairment and functional impairment are present during the episode of depression (Kim et al., 2021), and patients with comorbidity would display greater impairment than patients with either depression or a chronic disease exclusively (Moussavi et al., 2007; Almeida et al., 2020). Additionally, patients with depression symptoms can reduce compliance to treatment, which might lead to poor disease prognosis or even death (Grenard et al., 2011; Kistler et al., 2022).

There is extensive evidence indicating that a huge variety of factors contribute to depression in the elderly population. A recent systematic review (Maier et al., 2021) including 30 longitudinal studies summarized the risk factors of depression, including genetical factors, sociodemographic and relationship characteristics, lifestyle, mental health, physical health, impairment, and psychosocial factors. A Brazil population-based study reported the unequally distributed in population: it is higher in women, older individuals, widowed or divorced, and poor ones (Boing et al., 2012). These results were consistent with the studies in Europe and China (Conde-Sala et al., 2019; Kim et al., 2021; Ye et al., 2021). However, some heterogeneous results were found under different populations. For example, some studies indicated that rural residence was a risk factor for depression in Nigeria (Gureje et al., 2011) and China (Gu et al., 2013; He et al., 2020; Yan et al., 2023), but not in Japan (Misawa and Kondo, 2019). In factors related to lifestyle, unhealthy behaviors such as smoking and alcohol consumption (Kim et al., 2021) and poor sleep quality (Amelia et al., 2022) may induce depressive symptoms, while mental health benefits from being physically active (Pearce et al., 2022). Concerning psychosocial factors, the absence of family or social support is also a significant risk factor for poor rehabilitation and prevention of depression (Compare et al., 2013; Abraham et al., 2022). Beyond that, personality traits such as neuroticism/negative emotionality increase the risk of depression in the face of negative life events (Klein et al., 2011).

Despite the important relationship between chronic diseases and depression, there are scarce Chinese population evidence studies, especially the exploration of risk and protective factors of depression in patients suffering chronic diseases. Moreover, the subjects in many existing empirical studies (e.g., Boing et al., 2012; Liu H. et al., 2023) were hospitalized patients or older adults. While he former one did not include outpatients, the latter one included those with no chronic disease, and these may be somewhat biased in representing the patients with chronic disease and estimating the relation with depression. In this study, we focus on the patients with chronic disease, and drew subjects from a nationally representative study of Chinese community-dwelling adults.

In the present study, we conducted an empirical study based on data obtained from CHARLS to explore the current status of depression and its influencing factors among middle-aged and elderly patients with chronic diseases in China. Reviewing literatures concerned, we developed a conceptual framework for risk factors for depression from six dimensions, including demographic characteristics, sociological characteristics, economic characteristics, physical health status, lifestyle, and subjective satisfaction. Given the accessibility of measures in the database, a number of candidate predictors of each dimension were selected for data analysis (Table 1). The results of this study help in improving the mental health status of the patient with chronic diseases and optimize care of patients with comorbid depressive disorder and physical disease.

**Table 1:** Variable assignment table of depressive factors in elderly patients with chronic diseases.

Dimension	Variable	Values
Dependent variable	Depressive symptoms	0 = no, 1 = yes
Demographic characteristic	Age	Measured value
	Sex	0 = male, 1 = female
	Education level	1 = illiterate, 2 = below primary school, 3 = primary school, 4 = junior high school, 5 = senior high school, 6 = junior college and above
	Location	0 = rural, 1 = urban
Sociological characteristics	Whether living with spouse	0 = no, 1 = yes
	Whether living with children	0 = no, 1 = yes
	Number of children	Measured value
	Frequency of child’s visit	Measured value (proportion of non-living children who visit at least once a month)
	Frequency of contacting with children	Measured value (proportion of children who do not live together who contact each other at least once a month)
	Alimony	Measured value (taking logarithm)
	Whether taking care of grandchildren	0 = no, 1 = yes
Economic characteristics	Whether having pension insurance of government public institution	0 = no, 1 = yes
	Whether having residents’ basic pension insurance	0 = no, 1 = yes
	Whether having life insurance or commercial insurance	0 = no, 1 = yes
	Whether having medical insurance	0 = no, 1 = yes
	Whether having non-farm jobs other than self-employment	0 = no, 1 = yes
	Whether retirement	0 = no, 1 = yes
	Wage bonus subsidy	Measured value (taking logarithm)
	Provident fund income	Measured value (taking logarithm)
	Pension	Measured value (taking logarithm)
	Existing debts	Measured value (taking logarithm)
Physical health status	Chronic disease situation	0 = single，1 = multiple
	Self-rated health	1 = very bad, 2 = bad, 3 = fair, 4 = good, 5 = very good
	Whether disability	0 = no, 1 = yes
	Vision - farsightedness	1 = bad, 2 = fair, 3 = good, 4 = very good, 5 = excellent
	Vision - Myopia	1 = bad, 2 = fair, 3 = good, 4 = very good, 5 = excellent
	Hearing	1 = bad, 2 = fair, 3 = good, 4 = very good, 5 = excellent
	Pain and discomfort situation	1 = never, 2 = almost never, 3 = sometimes, 4 = fairly often, 5 = very often
	BADL impairment	0 = no, 1 = yes
	IADL impairment	0 = no, 1 = yes
Lifestyle	Sleep time	Measured value
	High intensity of physical activity	Calculated value by formula
	Moderate intensity of physical activity	Calculated value by formula
	Mild intensity of physical activities	Calculated value by formula
	Breadth of social activities	Measured value (types of social activities)
	Intensity of social activities	Measured value (time spent in social activities)
	Whether smoking	0 = no, 1 = yes
	Whether drinking	0 = no, 1 = yes
Subjective satisfaction	Life satisfaction	1 = not at all satisfied, 2 = not very satisfied, 3 = somewhat satisfied, 4 = very satisfied, 5 = extremely satisfied
	Marital satisfaction	1 = not at all satisfied, 2 = not very satisfied, 3 = somewhat satisfied, 4 = very satisfied, 5 = extremely satisfied
	Parent–child relationship satisfaction	1 = not at all satisfied, 2 = not very satisfied, 3 = somewhat satisfied, 4 = very satisfied, 5 = extremely satisfied
	Air quality satisfaction	1 = not at all satisfied, 2 = not very satisfied, 3 = somewhat satisfied, 4 = very satisfied, 5 = extremely satisfied

## 2 Materials and methods

### 2.1 Study subjects

In this study, the data was drawn from the 2018 wave of the CHARLS. This database conducted surveys in 150 districts, 450 villages and units, and tens of thousands of households in 28 provinces (municipalities and autonomous regions) across the country. As per the research aim, middle-aged and elderly patients aged ≥45 years and suffering from chronic diseases were selected as the analysis sample. Individuals were excluded if there were severe missing data for the selected variables. At the same time, a multiple imputation method was employed to compute any small amount of missing data in a depression scale. The study finally identified 6,704 valid samples. Among these patients, 14 different types of chronic diseases were recorded, namely, hypertension, dyslipidemia, diabetes, cancer and other malignant tumors, chronic pulmonary diseases, liver diseases, heart diseases, stroke, kidney diseases, stomach diseases or digestive system diseases, emotional and mental problems, memory-related diseases, arthritis or rheumatism, and asthma. There were 4,246 valid samples with a single chronic disease, and 2,458 with multiple chronic diseases. The study design was approved by the Biomedical Ethics Committee of Peking University (IRB00001052-11015), and an informed consent form was signed with each respondent.

### 2.2 Research methods

“Depressive symptoms” served as the dependent variable in this study. The CHARLS project used the simplified version of the Center for Epidemiologic Studies Depression Scale (CES-D) revised by Andresen et al. (1994). The scale scored depression from 0 to 30 points where higher score indicates higher degree of depression. A score ≥ 10 was indicative of depressive symptoms (Boey, 1999). The Cronbach’s alpha for the CES-D simple scale was 0.813, which indicates the high reliability of the scale.

Considering the previous relevant studies and the accessibility in the publicly database, we chose six dimensions of influencing factors, namely, demographic characteristics (including age, sex, education level, location), sociological characteristics (including whether living with spouse, number of children, living or contacting situation with children, alimony, whether taking care of grandchildren), economic characteristics (including pension insurance, medical insurance, having work other than agricultural self-employment, retired, work income, existing debt), physical health status (including chronic disease situation, self-rated health, disability, vision and hearing, pain and discomfort situation, physical function impairment), lifestyle (including sleep time, physical activity level, social activity situation, smoking, drinking), and subjective satisfaction (life satisfaction, marital satisfaction, children relationship satisfaction, air quality satisfaction). In total, 42 influencing factors were analyzed. The specific variable assignment is shown in Table 1.

### 2.3 Statistical methods

Continuous variables were described as mean ± standard deviation, and group comparison was performed using an independent sample *t*-test. Categorical variables were expressed as frequency and percentage, and group comparison was performed using a *χ*^2^ test. For ordered categorical independent variables with normal distribution, such as the Likert scale variables of life satisfaction in Table 1, no dummy variable setting was done. These were treated as continuous variables, which would facilitate subsequent model analysis and result interpretation.

For predictive fitting model, a LASSO-logistic regression model was employed to identify key indicators that influenced the depressive symptoms in this cohort. The LASSO method offers the advantage of adding a penalty term, and directly compresses the regression coefficients of unimportant variables to zero. The correlations coefficients among the studied variables ranged from 0.002 to 0.651. In addition, some predictors had a variance inflation factors (VIF) of above 10, indicating the multicollinearity between predictors. Therefore, we used LASSO method for the purpose of variable selection and to avoid problems such as overfitting and multicollinearity. Additionally, a nomogram plot was generated for visualization of complex regression model results.

For model evaluation and validation, the area under the receiver operating characteristic (ROC) curve (AUC) was used to evaluate the model discrimination, and Hosmer-Lemeshow goodness-of-fit test and calibration curve were employed to assess the model calibration. To reasonably evaluate the generalization ability of the model, 70% of the samples were randomly extracted to form the modeling group population (*N* = 4,693), and the remaining 30% of the samples were used as the validation group population (*N* = 2011). This study used R language software for data analysis. LASSO-logistic regression analysis was performed using the glmnet package, and nomogram plot was drawn using the rms package.

## 3 Results

### 3.1 Basic situation

We analyzed 6,704 middle-aged and elderly patients with chronic diseases; their mean age was 61.62 (*SD* = 9.37) years, and 3,101 (42.3%) were male. Overall, 3,783 (56.4%) patients were at least 60 years old, 5,011 (74.7%) lived in rural areas, and 3,677 (54.8%) had at least high school level of education. The most commonly reported chronic disease was hypertension (34.0%), followed by dyslipidemia (25.7%). The specific demographic characteristics and distribution of various chronic diseases are shown in Table 2.

**Table 2:** Descriptive statistics of demographic characteristics.

Variable	Percentage	Variable	Percentage
Age		Chronic diseases	
45 ~ 49	9.8%	Hypertension	34.0%
50 ~ 59	33.8%	DyslipIdemia	25.7%
60 ~ 69	35.4%	Diabetes	13.3%
≥ 70	21.0%	Cancer and other malignant tumors	2.8%
Sex		Chronic lung disease	12.6%
Male	46.3%	Liver disease	7.7%
Female	53.7%	Heart disease	18.4%
Education level		Apoplexy	10.5%
Illiterate	22.6%	Kidney disease	9.6%
Below primary school	0.06%	Stomach or digestive disorders	23.4%
Primary school	22.3%	Emotional and spiritual problems	2.1%
Junior high school	0.24%	Memory related diseases	3.9%
Senior high school	22.6%	Arthritis or rheumatism	25.6%
Junior college and above	32.2%	Asthma	4.8%
Location		Depressive symptoms	
Rural	74.7%	No	54.4%
Urban	25.3%	Yes	45.6%

The mean CES-D score for depression was 8.64 (*SD* = 6.68), and 3,058 (45.6%) patients showed depressive symptoms. Among the population with depressive symptoms, 1,906 (52.9%) were female who had much higher depression level than the male patients (37.1%); 1,785 (42.0%) patients had a single chronic disease, and 1,273 (51.8%) had multiple chronic diseases; 370 (72.8%) patients had BADL impairment and 947 (65.2%) showed IADL impairment. In comparison with the population without depressive symptoms, that with depressive symptoms showed significantly lower mean self-rated health value, higher pain and discomfort, shorter average sleep time, lower education level, more children but lower visit frequency, lower light physical activity but higher moderate and high intensity physical activity level, lower social activity breadth and depth, lower alimony and income, lower life, marriage, or children relationship, and lower air quality satisfaction scores (*p*s < 0.05). The specific variable data and related tests are shown in Tables 3, 4.

**Table 3:** Data set categorical (count) variables.

Variable	Group without depression (*N* = 3,646)	Group with depression (*N* = 3,058)	*χ* ^2^	*p*	Variable	Group without depression (*N* = 3,646)	Group with depression (*N* = 3,058)	*χ* ^2^	*p*
Sex			166.04	<0.001	Medical insurance			6.189	0.013
Male	1949	1,152			No	78	96		
Female	1,697	1906			Yes	3,568	2,962		
Location			101.78	<0.001	Non-farm jobs other than self-employment			50.27	<0.001
Rural	2,546	2,465			No	2,735	2,514		
Urban	1,100	593			Yes	911	544		
Live with spouse			59.10	<0.001	Retirement			74.72	<0.001
No	609	743			No	3,146	2,840		
Yes	3,037	2,315			Yes	500	218		
Live with child			2.44	0.118	Chronic diseases			59.27	<0.001
No	2,481	2025			Single	2,461	1785		
Yes	1,165	1,033			Multiple	1,185	1,273		
Care for grandchild			0.60	0.439	BADL impairment			162.98	<0.001
No	2,753	2,283			No	3,508	2,688		
Yes	893	775			Yes	138	370		
Government and public institutions’ pension insurance			126.88	<0.001	IADL impairment			286.18	<0.001
No	2,927	2,759			No	3,141	2,111		
Yes	719	299			Yes	505	947		
Residents’ basic pension insurance			56.87	<0.001	Smoking			38.83	<0.001
No	1,186	738			No	2,594	2,381		
Yes	2,460	2,320			Yes	1,052	677		
Life or commercial insurance			15.68	<0.001	Drinking			81.86	<0.001
No	3,385	2,911			No	2,304	2,250		
Yes	261	147			Yes	1,342	808		

**Table 4:** Mean comparison of continuous type (measurement) variables.

Variable	Group without depression	Group with depression	*t*	*p*
Age	61.77	62.27	−2.27	0.024
Education level	4.36	3.68	15.56	<0.001
Number of children	1.36	1.58	−5.89	<0.001
Frequency of child’s visit	0.13	0.12	2.10	0.036
Frequency of contacting with children	0.22	0.22	0.66	0.508
Alimony	2.05	1.73	3.87	<0.001
Wage bonus subsidy	2.46	1.59	9.93	<0.001
Provident fund income	0.17	0.08	4.05	<0.001
Pension	4.66	4.26	4.25	<0.001
Existing debts	0.58	0.72	−2.16	0.031
Self-rated health	3.02	2.43	26.30	<0.001
Vision – farsightedness	2.33	1.92	17.60	<0.001
Vision – Myopia	2.31	1.97	15.33	<0.001
Hearing	2.47	2.16	13.80	<0.001
Pain and discomfort situation	2.08	2.90	−25.65	<0.001
Sleep time	6.45	5.60	17.53	<0.001
High intensity of physical activity	2138.48	2373.42	−3.99	<0.001
Moderate intensity of physical activity	1343.59	1381.45	−1.31	<0.001
Mild intensity of physical activities	1997.06	1944.41	2.15	0.032
Breadth of social activities	1.09	0.83	9.47	<0.001
Intensity of social activities	2.16	1.58	9.94	<0.001
Life satisfaction	3.42	2.90	26.51	<0.001
Marital satisfaction	3.28	2.78	16.17	<0.001
Parent–child relationship satisfaction	3.73	3.46	14.50	<0.001
Air quality satisfaction	3.23	3.05	8.66	<0.001

### 3.2 Variable selection

**Figure 1:**
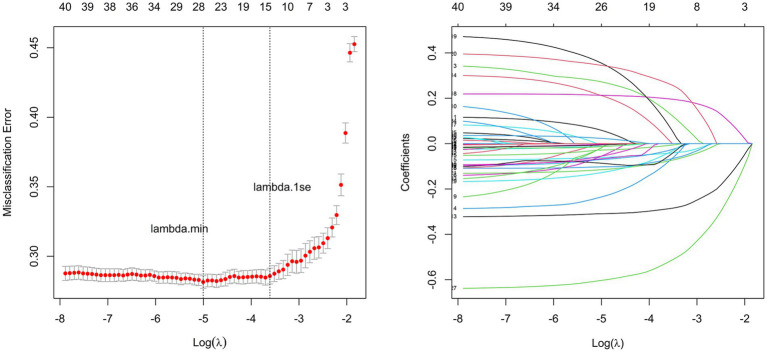
Variable selection based on LASSO regression. The subfigure on the left shows the selection process of the value of lambda by cross validation. The subfigure on the right shows selection process of the coefficients of variables by LASSO regression.

Based on the modeling group data, we performed LASSO-logistic regression to reduce the dimension of the 42 candidate variables and preliminarily screen the predictive factors of depression in this cohort. To obtain a model with good performance, a 10-fold cross-validation method was used and the value of the minimum cross-validation error served as the optimal penalty parameter. As shown in Figure 1, the optimal parameter *λ* was 0.0067 (i.e., log*λ* = 5.004). There were 26 variables with non-zero regression coefficients, including gender, residence, education level, whether living with spouse, visits by children, alimony, government or public institution pension insurance, life insurance or commercial pension insurance, etc., retired, salary bonus subsidy, pension, existing debt, chronic diseases, self-rated health, disabled, vision-farsightedness, vision-nearsightedness, pain and discomfort, BADL impairment, IADL impairment, sleep time, social activity depth, life satisfaction, marital satisfaction, children relationship satisfaction, air quality satisfaction.

### 3.3 Model construction and nomogram

Considering the bias in the parameter estimation by the LASSO method (Fan and Li, 2001), we further selected 26 important factors as independent variables and constructed a multifactorial Logistic regression analysis model by considering depressive symptoms as a dependent variable to determine the final predictive factors and generate a nomogram. At a significance level of 10%, gender, residence, education level, whether living with spouse, visits by children, alimony, pension, current debt, chronic diseases, self-rated health, disability, vision myopia, pain-related discomfort, BADL impairment, IADL impairment, sleep time, social activity depth, life satisfaction, marital satisfaction, children’s relationship satisfaction, and air quality satisfaction were the 21 variables that affected depression in our study cohort (Table 5).

**Table 5:** Multifactorial Logistic regression analysis results of depression in middle-aged and elderly patients with chronic diseases.

Variable	*β*	OR (95%CI)		*p*
Sex	0.320^***^	1.377	(1.188, 1.597)	<0.001
Location	−0.295^**^	0.745	(0.623, 0.890)	0.001
Education level	−0.068^**^	0.934	(0.897, 0.972)	0.001
Whether living with spouse	−0.144^†^	0.866	(0.706, 1.063)	0.068
Frequency of child’s visit	−0.148^†^	0.863	(0.627, 1.185)	0.064
Alimony	−0.019^†^	0.981	(0.961, 1.002)	0.072
Whether having pension insurance of government public institution	−0.071	0.932	(0.697, 1.243)	0.633
Whether having life insurance or commercial insurance	−0.148	0.862	(0.647, 1.145)	0.309
Whether retirement	−0.089	0.915	(0.648, 1.290)	0.613
Wage bonus subsidy	−0.013	0.987	(0.968, 1.006)	0.168
Pension	−0.026^*^	0.975	(0.955, 0.995)	0.013
Existing debts	0.039^**^	1.039	(1.012, 1.067)	0.004
Chronic disease situation	0.124^*^	1.132	(0.984, 1.302)	0.043
Self-rated health	−0.315^***^	0.730	(0.671, 0.794)	<0.001
Whether disability	0.282^**^	1.325	(1.102, 1.595)	0.003
Vision - farsightedness	−0.034	0.967	(0.890, 1.050)	0.424
Vision - myopia	−0.084^*^	0.919	(0.846, 0.999)	0.047
Pain and discomfort situation	0.219^***^	1.244	(1.177, 1.316)	<0.001
BADL impairment	0.463^**^	1.588	(1.190, 2.130)	0.002
IADL impairment	0.378^***^	1.460	(1.214, 1.755)	<0.001
Sleep time	−0.131^***^	0.878	(0.848, 0.908)	<0.001
Intensity of social activities	−0.011^†^	0.989	(0.960, 1.019)	0.078
Life satisfaction	−0.635^***^	0.530	(0.479, 0.586)	<0.001
Marital satisfaction	−0.106^**^	0.899	(0.840, 0.963)	0.002
Parent–child relationship satisfaction	−0.165^**^	0.848	(0.767, 0.938)	0.001
Air quality satisfaction	−0.096^*^	0.909	(0.835, 0.990)	0.028

^***^, ^**^, ^*^, and ^†^ Indicate significance at 0.1, 1, 5, and 10% levels, respectively.

**Figure 2:**
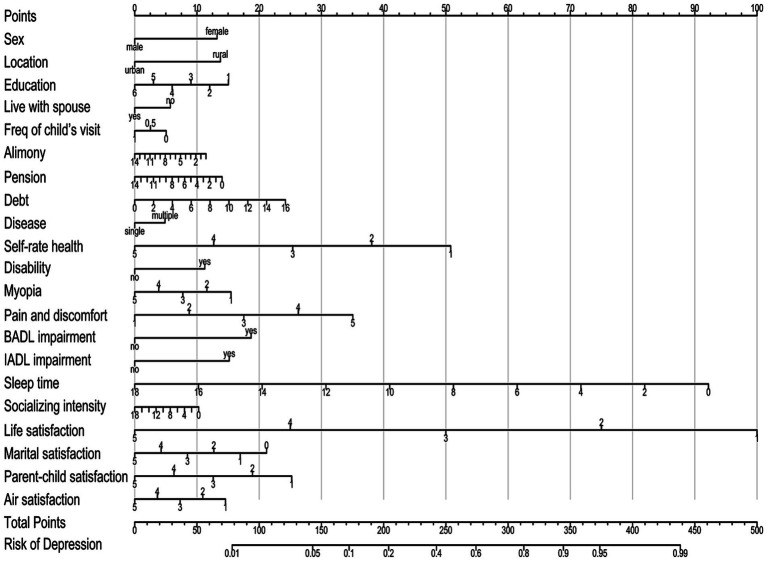
Nomogram for predicting depression risk in middle-aged and elderly patients with chronic diseases.

We included these influencing variables screened out by the multifactorial Logistic regression analysis in the nomogram prediction model. As shown in Figure 2, the score values for all influencing variables are projected vertically to the top score axis and summed to obtain a total score. The corresponding depression occurrence risk probability is then obtained from the vertical projection of the total score line on the bottom risk axis.

### 3.4 Model evaluation and validation

**Figure 3:**
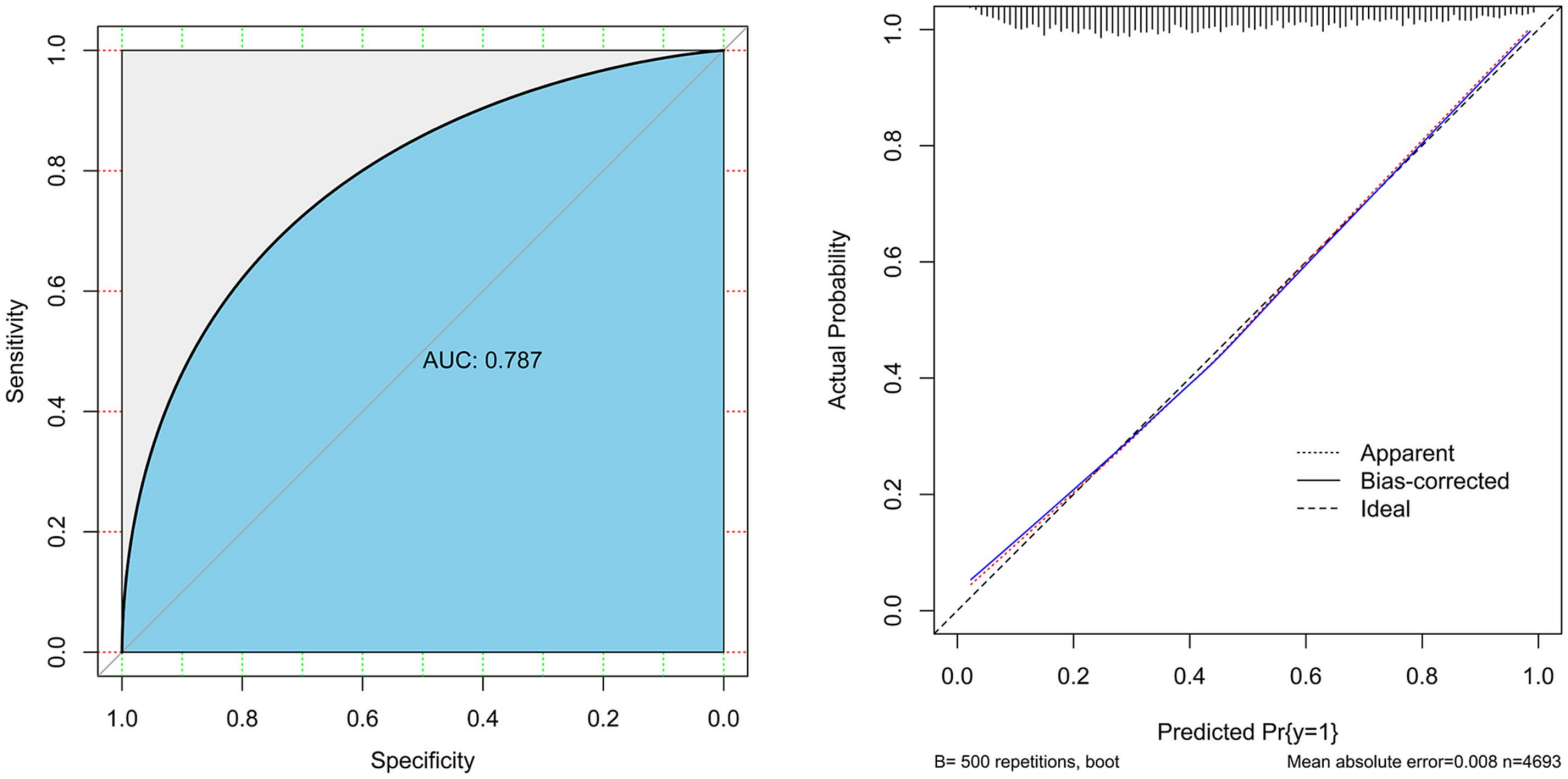
Discrimination and calibration tests of the fitted model based on LASSO-logistic regression. The subfigure on the left is the ROC curve, and the subfigure on the right is the calibration curve.

In the modeling group, we generated a ROC curve (Figure 3, left) and found that the prediction model had an AUC value of 0.788, specificity of 72.8%, and sensitivity of 71.0%, which indicates that the model had good discrimination. The Hosmer-Lemeshow goodness-of-fit test result (*χ*^2^ = 11.39, *df* = 8, *p* = 0.181) also revealed that the model had good goodness-of-fit. The Bootstrap method was used to perform internal validation with 500 resampling times. The results showed good repeatability for the model’s standard curve and actual curve (Figure 3 right), with an average absolute error of 0.008. Thus, the model showed good calibration. In the validation group, the AUC of the model was 0.783, and its specificity and sensitivity were 70.8, and 71.6%, respectively. The Hosmer-Lemeshow goodness-of-fit test indicated its good fit (*χ*^2^ = 10.23, *df* = 8, *p* = 0.249), while the calibration plot demonstrated the good consistency between predicted results and observed results. Therefore, the model showed good discrimination and calibration.

## 4 Discussion

The results established herein showed that different demographic characteristics, such as female sex, living in rural areas, and low education were the risk factors associated with depression among our study cohort, which was consistent with existing study results in China and worldwide (Russell and Taylor, 2009; Li et al., 2022; Als et al., 2023). According to the CHARLS 2018 survey data, middle-aged and elderly women from China showed more severe depressive symptoms (accounting for 43.6%) (Ye et al., 2021) and the incidence was 2–3 times higher than that in men, while middle-aged and elderly women with chronic diseases had more prominent depression (accounting for 52.9%). Differences in depressive symptoms were also observed between urban and rural dwellers, which is consistent with some longitudinal studies of the same cohort (He et al., 2020; Yan et al., 2023). In addition to the excessive pressure that the modern society imposes on women, personality, genetics, some substances in the body, and metabolic disorders are important causes of high incidence of depression in women (Russell and Taylor, 2009; Mei et al., 2019; Als et al., 2023). After controlling other influencing variables, the risk of depressive symptoms in subjects dwelling in urban areas was found to be 0.745 times that reported in those dwelling in rural areas. Thus, urban resources can effectively alleviate depression among this patient population. Senior intellectuals are thought to be more prone to depression, but the nomogram constructed in this study showed that those with high education have a lower risk of depression. Some studies have pointed out that education may have an age effect on depression (Galambos et al., 2006; Li et al., 2022), and can show different patterns and characteristics during the course of an individual’s life cycle (Kamiya et al., 2013).

Sociological and economic characteristics were associated with depressive symptoms in our study cohort. These include whether living with spouse, visits by children, alimony, pension, and current debt. In the depression-susceptible population without spouse and poor physical health, family support can effectively buffer the impact of adverse situations and exert beneficial effects by alleviating depression, which is consistent with previously published results (Wang et al., 2006; Russell and Taylor, 2009; Ye et al., 2021). Middle-aged and elderly patients with chronic diseases face the dual pressure of health and economy. Economic welfare is an important determinant of their quality of life as well as physical and mental health. Considering the degree of influence of each economic factor in the nomogram, one can speculate that the support of children’s alimony and social pension insurance is relatively limited. The sense of security bestowed by good personal savings and no debt burden is an effective way to reduce the occurrence of depression, which is consistent with the existing research results (Choi and Jun, 2009; Liu J. et al., 2023).

Depression in this patient cohort was closely related to physical health and lifestyle, including having multiple chronic diseases, poor self-rated health, disability, myopia vision impairment, pain-related discomfort due to illness, BADL and IADL impairment, short sleep time, and lack of social activities. This patient population has limited daily activity owing to physical health impairment, which may seriously affect their mental health and contribute to depressive symptoms (Chen et al., 2020; Wang et al., 2023). This serves as a reminder to the medical staff and community workers to focus on strengthening health education, as well as paying attention to the mental health of these patients. Growing number of elders with mobility impairment disorders, is expected to increase the investment in assistive devices such as wheelchairs, walkers, and reading glasses. Furthermore, recommendation can be made for the participation in appropriate social activities such as square dancing and mountain climbing, to reduce the negative impact of physical illness on mental health.

In our study, depression negatively correlated with subjective satisfaction and was particularly associated with life satisfaction, followed by children’s and marital satisfaction. Good intergenerational relationships and stable marital relationships have a significant positive effect on the life of middle-aged and elderly groups (Deng and Zhang, 2019), which in turn directly affects their mental health and reduces the risk of depression (Abraham et al., 2022; Deng et al., 2023).

In summary, depressive symptoms in middle-aged and elderly patients with chronic diseases are caused by multiple factors. First, we propose that this patient population should actively seek treatment for chronic diseases, strengthen pain management, improve sleep quality, meet and contact with their children frequently, participate in social activities and pension insurance, form rational consumption and saving habits, and enrich their later life. Second, children should continue to carry forward the Chinese filial culture and play the role of material support and spiritual comfort to their elders. Third, primary medical and public health institutions should pay more attention while nursing toward those who are at a high risk of depression by providing psychological counseling and appropriate care and improve their mental health. Fourth, measures should be directed to improve and optimize the social pension security system, given the positive effects of pension insurance, medical security, and social interaction on the life quality of this patient population.

This study has some limitations. First, the current study design relies on cross-sectional data, which is correlational in nature and do not imply causation. Further analysis based on longitudinal follow-up surveys is warranted. Second, our sample was Chinese and thus generalizability across racial and ethnic groups should not be assumed. Last, the dataset was limited in the available measures of some plausible predictors such as personality traits and mental health, which hindered us further exploring the interplay of personality traits and depression among the patients with chronic diseases.

## Data availability statement

The original contributions presented in the study are included in the article/supplementary material, further inquiries can be directed to the corresponding authors.

## Ethics statement

The studies involving humans were approved by the Biomedical Ethics Committee of Peking University. The studies were conducted in accordance with the local legislation and institutional requirements. The participants provided their written informed consent to participate in this study.

## Author contributions

WL: Methodology, Project administration, Writing – original draft, Writing – review & editing. DZ: Investigation, Project administration, Writing – original draft, Writing – review & editing. YW: Conceptualization, Investigation, Methodology, Writing – review & editing. LZ: Funding acquisition, Investigation, Writing – review & editing. JY: Data curation, Formal analysis, Resources, Writing – original draft, Writing – review & editing.
